# Stochastic and Deterministic Models of Cellular p53 Regulation

**DOI:** 10.3389/fonc.2013.00064

**Published:** 2013-04-02

**Authors:** Gerald B. Leenders, Jack A. Tuszynski

**Affiliations:** ^1^Department of Physics, University of AlbertaEdmonton, AB, Canada

**Keywords:** p53, cell cycle, cancer, stochastic modeling, deterministic modeling, desynchronization

## Abstract

The protein p53 is a key regulator of cellular response to a wide variety of stressors. In cancer cells inhibitory regulators of p53 such as MDM2 and MDMX proteins are often overexpressed. We apply *in silico* techniques to better understand the role and interactions of these proteins in a cell cycle process. Furthermore we investigate the role of stochasticity in determining system behavior. We have found that stochasticity is able to affect system behavior profoundly. We also derive a general result for the way in which initially synchronized oscillating stochastic systems will fall out of synchronization with each other.

## Introduction

Among the vast number of mechanisms utilized by cancer cells to sustain cell division, the inactivation of the essential tumor suppressor and transcription factor p53 is one of the most frequent and effective strategies. Therefore, restoring the activity of the p53-signaling pathway is currently one of the most promising therapeutic strategies for fighting this disease (Levine and Oren, 2009).

In normal cells, p53 plays a central role in the regulation of the cell cycle, apoptosis, DNA repair, and senescence (Teodoro et al., 2007); p53 responds to cellular stress, such as hypoxia or DNA damage, by accumulating in the nucleus, regulating the expression of target genes, and activating/inactivating various pathways in order to maintain the normal function of the cell (Maltzman and Czyzyk, 1984; Kastan et al., 1991; Graeber et al., 1994). Indeed, it appears that whenever the integrity of a cell’s genetic code is threatened, p53 is there to protect it. This conclusion has led p53 to be called the guardian of the genome (Lane, 1992).

However, the p53-signaling pathway is inoperative in almost all types of human cancer; factors that inactivate p53 specifically include genetic mutations or deletions (Feki and Irminger-Finger, 2004), defective post-translational modifications, and interactions with its main endogenous inhibitors, MDM2 (Momand et al., 1998) and MDMX (Shvarts et al., 1996). Excitingly, a number of these tumors have been shown to have a less invasive phenotype upon restoration of p53 activity (Olivier et al., 2002; Ventura et al., 2007; Suad et al., 2009; Mandinova and Lee, 2011).

With the cost of drug development on the scale of hundreds of millions to billions of dollars per new drug entity – and rising – there is strong need to look for any possible acceleration and improvement to the efficiency and accuracy of the development process (Paul et al., 2010). Thanks to the increasing computing power available to researchers, it is now becoming affordable and practical to attempt to use *in silico* models to improve the development process. One way to do this is to improve the ability of researchers to select appropriate proteins, or interactions between proteins, as targets for drug development by better understanding their function in protein interaction networks.

The purpose of this study is to gain new insights into the functioning of p53, a central protein in cell cycle regulation. A simple model of p53 oscillations in response to ionizing radiation is presented. Additionally, the behavior of stochastic and deterministic representations of the same model system is compared.

### Cell cycle

The protein p53 is a regulator of the cell cycle and cell fate. Under normal conditions, a cell will normally progress through several stages. In the G1 phase (first gap phase) the cell grows in size to prepare for DNA synthesis. After G1, the cell moves into S phase (synthesis phase), during which new DNA is synthesized. Cells that are not replicating can also leave G1 and enter the G0 phase, a state in which they do not grow, and can remain quiescent indefinitely. Next comes the G2 phase (second gap phase), where cells grow further and complete their final preparations for mitosis. Mitosis then occurs and the cycle can begin anew (Lodish et al., 2008). A damaged cell may need to halt its cycle or even self-destruct in a process called apoptosis. Apoptosis is necessary for normal development and homeostasis of multicellular organisms, and is also a desirable outcome for cancer cells during cancer chemotherapy.

**Figure 1:**
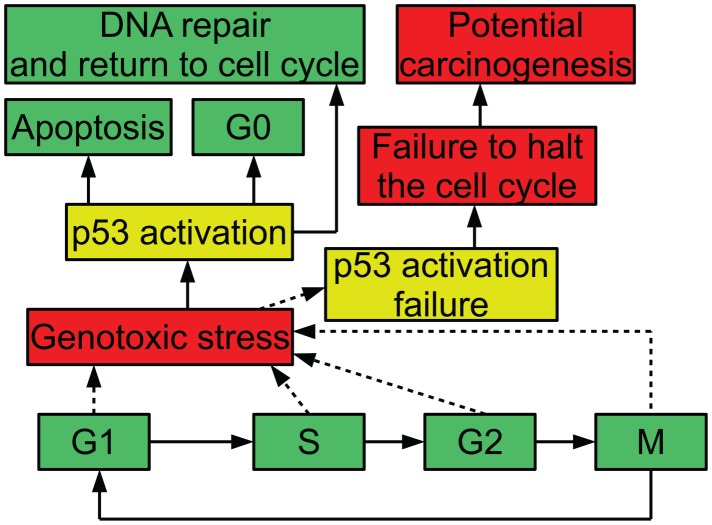
**Diagram of p53 and the cell cycle, showing possible outcomes of stress and p53 activation**.

In order to ensure that the process of cell division is carefully regulated, the cell has a number of checkpoints. These checkpoints are conditions that a cell must meet in order to progress in the cell cycle. For example, one checkpoint in G1 ensures that a cell has grown sufficiently in size to move into S phase and replicate its DNA. Another checkpoint that occurs in G1 is mediated by the protein p53: when DNA is damaged, p53 halts the cell cycle until the damage is repaired; this prevents the cell from trying to duplicate the damaged DNA. When p53 is inactivated, this checkpoint no longer functions. A cell attempting to duplicate damaged DNA is likely to accumulate mutations (Alberts et al., 1994). Figure 1 diagrams the relevance of p53 to the cell cycle.

### p53

The protein p53 responds to many stressors including ultraviolet light (Maltzman and Czyzyk, 1984), ionizing radiation (Kastan et al., 1991), hypoxia, heat (Graeber et al., 1994), improper cell adhesion (Nigro et al., 1997), ribonucleotide depletion (Linke et al., 1996), and infection by influenza (Turpin et al., 2005). Some viral proteins are known to interact with p53, for example hepatitis B virus HBx protein (Truant et al., 1995) and the large T antigen of simian virus 40 (Dobbelstein and Roth, 1998). The protein p53 has been demonstrated to induce cell cycle arrest, senescence, and apoptosis, with the specific outcome dependent on the extent and type of stress, and the genetic background of the cell (Vousden and Lu, 2002). The expression of p53 is tightly regulated by the cell (Sugrue et al., 1997; Lodish et al., 2008). In order to help it execute its various functions p53 is post-translationally modified at many sites to determine its response (Meek and Anderson, 2009; Dai and Gu, 2010). The protein p53 transcriptionally regulates numerous genes, with a pattern that varies depending on the type of stress and the cell type (Zhao et al., 2000). In addition to its transcriptional activity, p53 plays a transcription-independent role in apoptosis by binding to several anti-apoptotic proteins (Mihara et al., 2003).

The protein p53 is known to be mutated in approximately 50% of human tumors (Soussi and Wiman, 2007; Brown et al., 2009; Freed-Pastor and Prives, 2012). In addition, in tumors with wild type p53 it is common for p53 expression to be misregulated. For example, proteins that have a part in downregulating p53, such as MDM2 and MDMX, are commonly overexpressed in human tumors (Momand et al., 1998; Danovi et al., 2004). Furthermore, it has been demonstrated that restoration of p53 function can cause tumors to regress *in vivo* (Ventura et al., 2007). The importance of p53-signaling in cancer progression, and its therapeutic implications, have been investigated in previous mathematical models (Gammack et al., 2001; Perfahl et al., 2011), which highlights further our study.

Note that simply removing the limitations on a cell imposed by p53 is not enough for it to become cancerous; for a cell to become cancerous it must accumulate multiple hallmarks including: self-sufficiency in growth signals, insensitivity to anti-growth signals, limitless replicative potential, sustained angiogenesis, and the ability to migrate to other tissues (Hanahan and Weinberg, 2011). When such traits accumulate in a cell lacking functional p53, the probability of a cell becoming cancerous rises (Alberts et al., 1994).

### MDM2

**Figure 2:**
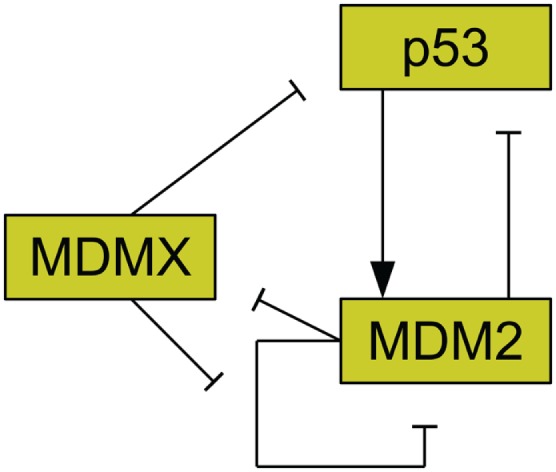
**Relationships between MDMX, MDM2, and p53**. MDM2 inhibits p53 and is promoted by it. MDM2 inhibits itself and this effect is reduced by MDMX. MDMX inhibits p53 directly, and is itself inhibited by MDM2.

The protein MDM2 is a key player in the regulation of p53 (Bond et al., 2005) and it has been found that MDM2 is commonly amplified in human cancers (Momand et al., 1998). MDM2 has been shown to be an E3 ubiquitin ligase for p53 (Honda et al., 1997). This means that MDM2 can mark p53 for degradation by the proteasome. As such, amplification of MDM2 leads to reduced p53 levels (Haupt et al., 1997; Kubbutat et al., 1997). MDM2 production is also induced by p53, forming a feedback loop (Barak et al., 1993). Figure 2 illustrates the interactions of MDM2 with p53. Additionally, MDM2 helps to regulate itself by autoubiquitination, meaning it marks itself for degradation by the proteasome (Fang et al., 2000). MDM2 possesses a nuclear localization signal, which is a structure on the protein that induces the cell to import the protein into the cell nucleus (Chen et al., 1995). MDM2 also has a cryptic nucleolar localization signal, which flags the protein for localization to the nucleolus, but only when MDM2 binding to another molecule changes the conformation of the signaling region (Lohrum et al., 2000).

In 2004 several small molecule inhibitors for the p53-MDM2 interaction were discovered (Vassilev et al., 2004). One of these inhibitors, Nutlin-3, was in Phase I clinical trials for retinoblastoma (Secchiero et al., 2011). Nutlins may also have some p53-independent effects, and these may be related to MDM2. It has been shown in some cell lines that MDM2 is upregulated by hypoxia independently of p53 (Gillespie, 2007). Furthermore, it has been shown that Nutlin-3 can radio-sensitize hypoxic cells that are p53 null, although it has a greater effect on cells with wild type p53 (Supiot et al., 2008). Additionally, Nutlin-3 has been shown to bind to several anti-apoptotic proteins other than MDM2, further complicating any analysis of its effects (Ha et al., 2011). MDM2 inhibitors bind to the protean competitively and occlude the binding site with p53 (Barakat et al., 2010). To the best of our knowledge Nutlins do not alter the autoubiquitination properties of MDM2.

### MDMX

Another important regulator of p53 is MDMX, a homolog of MDM2 (Shvarts et al., 1996; Finch et al., 2002). MDMX is commonly overexpressed in tumors, and its upregulation has been shown to promote tumor formation (Danovi et al., 2004). Unlike MDM2, however, MDMX expression is not induced by DNA damage (Shvarts et al., 1996). MDMX binds to both MDM2 (Sharp et al., 1999) and p53 (Shvarts et al., 1996). MDMX binding to MDM2 inhibits MDM2 autoubiquitination (Okamoto et al., 2009). Furthermore, MDM2 ubiquitinates MDMX (De Graaf et al., 2003). The interaction of MDMX and p53 has been shown to inhibit p53 activity (Marine et al., 2007). Figure 2 schematically depicts the relationships between p53, MDM2, and MDMX. MDMX possesses a cryptic nuclear localization signal (LeBron et al., 2006), so it can only reach the nucleus while bound to other proteins. MDMX is normally located primarily in the cytoplasm (Gu et al., 2002).

Small molecule inhibitors of MDMX have only recently been discovered (Reed et al., 2010). Although initial results show some efficacy against cancers with upregulated MDMX in cell culture (Wang et al., 2011), more work will need to be done to show whether or not they will be active *in vivo*, as well as whether or not it is the MDMX interaction or some off-target interaction that is causing the effect.

### Upstream regulators

There are many feedback loops known to affect p53, and the behavior of the p53 system is mediated by a number of upstream regulators (Harris and Levine, 2005). For example, the protein ATM is activated in response to ionizing radiation (Bakkenist and Kastan, 2003). Active ATM phosphorylates p53 (Banin et al., 1998), MDM2 (Maya et al., 2001), and Chk2 (Matsuoka et al., 2000). A related protein, ATR, phosphorylates p53 in response to single strand breaks in DNA (Tibbetts et al., 1999). Chk2 along with Chk1 also phosphorylate p53 (Shieh et al., 2000). These phosphorylations disrupt the ability of MDM2 to affect p53 (Zhang et al., 1998; Chehab et al., 2000; Maya et al., 2001).

### Other feedbacks

Aside from the MDM2 loop, there are other feedbacks affecting p53, although many of these involve also MDM2. The ARF protein is known to bind to MDM2 and promote its degradation (Zhang et al., 1998). ARF causes both MDM2 and MDMX to be localized to the nucleolus (Weber et al., 1999; Jackson et al., 2001). ARF is negatively regulated by p53 in a complex manner, thus forming a feedback loop (Stott et al., 1998; Lowe and Sherr, 2003). MDM2 activity becomes enhanced by a feedback in which p53 upregulates cyclin G, which then forms a complex with PP2A phosphatase. This complex then dephosphorylates MDM2, removing the inhibition caused by the phosphorylation effect (Harris and Levine, 2005). The Wip1 protein is induced by p53 and is able to modify ATM and Chk2, deactivating these proteins, and thus resulting in a stronger interaction between p53 and MDM2 (Fiscella et al., 1997; Fujimoto et al., 2006; Shreeram et al., 2006). Pirh2 has a more direct feedback with p53. Like MDM2, Pirh2 and COP1 both ubiquitinate p53 and are upregulated by p53 (Leng et al., 2003; Dornan et al., 2004).

### Protein level oscillations?

Lahav et al. (2004), Geva-Zatorsky et al. (2006), and Geva-Zatorsky et al. (2010) all directly observed sustained oscillations of p53 and MDM2 levels in the nuclei of individual cells. It is worth noting, however, that these single cell studies used MCF-7 cells. MCF-7 cells were initially used to study p53 because they exhibit wild type p53 (Lahav et al., 2004). Unfortunately, the MCF-7 cell line has a mutation in an MDM2 intron causing upregulation of MDM2 (Hu et al., 2007), lacks ARF (Stott et al., 1998), and possesses amplified MDMX (Danovi et al., 2004). Because of this, any assumption that any wild type cell would behave similarly to an MCF-7 cell with respect to p53 regulation is questionable at best. Unfortunately, there are no similar single cell studies of non-tumorigenic cell lines at the time of writing this paper. Also of note is the finding by Batchelor et al. (2011) that MCF-7 cells respond differently to damage induced by ultraviolet light than they do to double-strand breaks induced by gamma radiation or radiomimetic drugs. Geva-Zatorsky et al. (2006) also pointed out that undamped oscillations of p53 levels may appear damped in studies of cell populations due to the individual cells falling out of sync with each other. Damped oscillations have been observed in populations of non-tumorigenic cell lines, for example in entire mice (Hamstra et al., 2006).

### Previous modeling work

A number of models of p53 response to DNA damage have been proposed in the past. These models are based on a variety of approaches and serve a number of functions. Some basic models use built-in time delays on p53 induction of MDM2 transcription, such as some of the models developed by Geva-Zatorsky et al. (2006). In contrast, the model presented by Lev Bar-Or et al. (2000) used coupled differential equations to create time delay effects. There are advantages and disadvantages to each of these approaches. In a real cell, proteins are not produced instantly in response to a promoter. Both transcription and translation processes take time, and transport of the mRNA and the protein to the cytoplasm does not happen instantaneously. An explicit time delay deals with this problem directly, but may be more difficult to analyze than coupled equations. It also adds to the complexity of any computer algorithm made for stochastic simulations. A set of coupled equations, on the other hand, will start to show effects of induced protein production in the protein levels instantaneously, but the effect will be very small until some time has passed. In a stochastic system the protein levels are quantized and instead of instantaneous effects there is simply a small but non-zero possibility of instantaneous effects. In both the stochastic and deterministic cases adding more steps in the form of more coupled equations makes the system both more realistic and more computationally intensive. Another factor to consider is that p53 induces the transcription of MDM2 mRNA, and that mRNA is active for a time. Because of this, the actual rate of MDM2 production is dependent on a weighted average of past p53 levels rather than p53 levels at some specific time in the past. Using a single delayed p53 term to describe MDM2 production is therefore problematic. One way around this problem is to use a delay term for the production of the MDM2 mRNA rather than the MDM2 protein, as was done by Cai and Yuan (2009). Ma et al. (2005) investigated the number of p53 pulses that occur in response to DNA double-strand brakes using a model made from three linked modules, simulating DNA repair, ATM activation, and the p53-MDM2 feedback loop. Linking together multiple systems like this, in particular linking to systems that can be easily perturbed experimentally, may be a good way to develop models that are straight-forward to test. Batchelor et al. (2008) proposed a model based on abstracted signal and inhibitor systems interacting with MDM2 as well as active and inactive p53. This model was created to investigate the possible effects of ATM, CHK2, and WIP1 on p53 behavior. They included an equation for an input signal that converted p53 from an inactive form to an active form, and a p53 induced inhibitor that reduced the effects of the signal. There have also been past efforts to look at stochastic models of the p53 regulatory system. Cai and Yuan (2009) modeled p53-MDM2 and MDMX interactions and analyzed some of the effects of intrinsic noise. Their model has MDM2 mRNA being produced with a time delay. It also includes ubiquitinated states of proteins and a deubiquitination term, rather than just assuming all ubiquitinated proteins are degraded. Puszynski et al. (2008) developed a complex stochastic model of p53 behavior aimed at showing how stochastic effects lead to variability of cell fate in a bistable model. Their model includes a cytoplasmic compartment and a nuclear compartment, although p53 is not included in their cytoplasmic compartment. In addition to the negative feedback of MDM2 and p53 they include a positive feedback involving a series of events that lead to MDM2 being sequestered in the cytoplasm where it can no longer degrade p53.

Table 1 summarizes the key differences between the models. Ultimately, the differences in the models have as much, if not more, to do with differences in what the researchers were trying to investigate, rather than with differing assumptions about p53 behavior.

**Table 1:** **Key features of various models of p53 behavior**.

Model	Stochasticity	MDMX	Compartments	Time delayed equations	Stress signal	Other notes
Geva-Zatorsky et al. (2006)						These models do not have saturable MDM2 production
Model 1	Limited noise	No	No	No	No	Linear Model
Model 2	Limited noise	No	No	No	No	
Model 3	Limited noise	No	No	Yes	No	Linear Model
Model 4	Limited noise	No	No	No	No	
Model 5	Limited noise	No	No	No	No	Linear Model
Model 6	Limited noise	No	No	Yes	Yes	
Lev Bar-Or et al. (2000)	None	No	No	No	Yes	Stress is abstract and gets repaired
Ma et al. (2005)	In the stress and repair modules only	No	No	Yes	Yes	Complex stress and repair modules
Batchelor et al. (2008)	No	No	No	Yes	Yes	p53 promotes an inhibitor of the stress signal
Cai and Yuan (2009)	Yes	Yes	No	Yes	No	Includes phosphorylated proteins
Puszynski et al. (2008)	Yes	No	Yes, but not for p53	No	Yes	Includes many other proteins
Our model	Stochastic and non-stochastic versions were implemented	No	Only for MDM2	No	No	Details in Section “Materials and Methods”

## Materials and Methods

### The model

Since it has been observed that stochastic effects can cause a population of cells that undergo undamped oscillations to appear as if they were undergoing damped oscillations (Lahav et al., 2004; Geva-Zatorsky et al., 2006), it is interesting to compare a stochastic model of cell behavior to a deterministic one. By using both stochastic and deterministic versions of the same model it will be possible to look at the process of desynchronization between cells, which causes oscillations to appear damped, and to search for any other effects by which stochasticity could influence the system. As we shall see later, further investigation revealed several unexpected ways in which stochasticity influenced the system.

**Figure 3:**
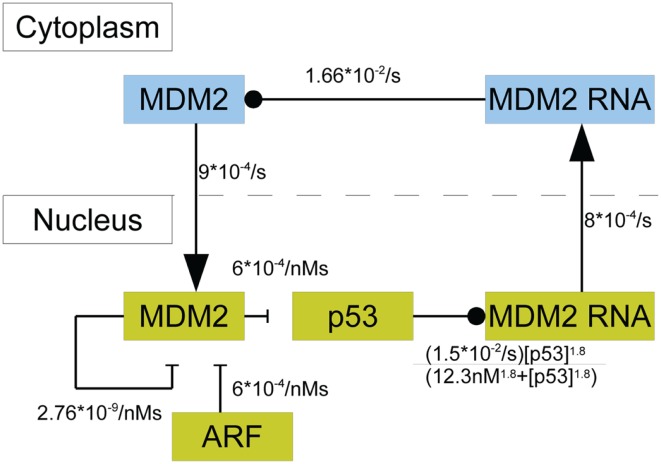
**A schematic of the model of p53 including MDM2 sequestration by ARF**. The blue boxes denote molecular species in the cytoplasm. The yellow boxes indicate molecular species in the nucleus. Arrows denote movement between compartments, barred lines indicate degradation, and circles indicate inducing production.

In this model p53 induces the transcription of MDM2 mRNA in the nucleus; there are three steps between induced transcription of MDM2 by p53 and the arrival of MDM2 proteins in the cell nucleus. Induced transcription is assumed to be proportional to $\left[\text{p}53\right]∕\left(K_{\text{D}}^{1.8}+{\left[\text{p}53\right]}^{1.8}\right),$ as was seen in the binding properties found by Weinberg et al. (2005). MDM2 mRNA is also produced at a basal rate. After being produced in the nucleus, the MDM2 mRNA proceeds to the cytoplasm, where it is translated and eventually decays. Even though mRNA from MDM2’s different promoter regions are translated at different rates, they are treated as one species. Because the two types of mRNA are assumed to decay at the same rate, this amounts to absorbing the difference in translation rates into the mRNA production rates. Cytoplasmic MDM2 moves to the nucleus at a constant rate, and all other behaviors that cytoplasmic MDM2 could exhibit are ignored in this model. ARF was given constant production and degradation rates. Once in the nucleus, MDM2 can become bound to ARF, which removes both proteins from the system. Additionally, MDM2 autoubiquitinates, which is a process that also removes it from the system. Figure 3 provides a schematic diagram of this system.

Using the principle of mass-action and the saturable transcription kinetics mentioned above, the system’s behavior can be mathematically described in terms of a system of differential equations. In addition to all the chemical reactions in Figure 3 the system of differential equations includes the production and degradation of p53, basal transcription of MDM2 mRNA, decay of cytoplasmic RNA, decay of ARF, and production of ARF. The equations are as follows:

$$\begin{array}{llll} & \frac{d\left[\text{p53}\right]}{dt}=k_{\text{p}}-k_{1}\left[\text{p53}\right]\left[\text{MDM}{\text{2}}_{\text{nuclear}}\right]-d_{\text{p}}\left[\text{p}53\right] & & \\ & \frac{d\left[\text{RN}{\text{A}}_{\text{nuclear}}\right]}{dt}=k_{\text{m}}\;+k_{2}\frac{{\left[\text{p53}\right]}^{1.8}}{k_{\text{D}}^{1.8}+{\left[\text{p53}\right]}^{1.8}}\;-k_{0}\left[\text{RN}{\text{A}}_{\text{nuclear}}\right] & & \\ & \frac{d\left[\text{RN}{\text{A}}_{\text{cytoplasmic}}\right]}{dt}=k_{0}\left[\text{RN}{\text{A}}_{\text{nuclear}}\right]-d_{\text{rc}}\left[\text{RN}{\text{A}}_{\text{cytoplasmic}}\right] & & \\ & \frac{d\left[\text{MDM}{\text{2}}_{\text{cytoplasmic}}\right]}{dt}=k_{\text{T}}\left[\text{RN}{\text{A}}_{\text{cytoplasmic}}\right] & & \\ & \;-k_{\text{i}}\left[\text{MDM}{\text{2}}_{\text{cytoplasmic}}\right] & & \\ & \frac{d\left[\text{MDM}{\text{2}}_{\text{nuclear}}\right]}{dt}=k_{\text{i}}\left[\text{MDM}{\text{2}}_{\text{cytoplasmic}}\right] & & \\ & \;-d_{\text{mn}}\left[{\text{MDM2}}_{\text{cytoplasmic}}^{\text{2}}\right] & & \\ & \;-k_{3}\left[\text{MDM}{\text{2}}_{\text{nuclear}}\right]\left[\text{ARF}\right] & & \\ & \frac{d\left[\text{ARF}\right]}{dt}=k_{\text{a}}\;-\;d_{\text{a}}\left[\text{ARF}\right]\;-\;k_{3}\left[\text{MDM}{\text{2}}_{\text{nuclear}}\right]\left[\text{ARF}\right] & & \end{array}$$

with *k*_p_ being the production rate of p53, *k*_1_ being the rate at which MDM2 ubiquitinates p53, and *d*_p_ being the rate of MDM2-independent p53 degradation. Here, *k*_m_ is the rate of p53-independent MDM2 mRNA production, *k*_2_ is the maximum rate of p53-dependent MDM2 mRNA production, *K*_D_ is the dissociation constant for p53 on the MDM2 promoter region, and *k*_0_ is the rate of MDM2 mRNA transport to the nucleus. In the equations above, *d*_rc_ is the decay rate of MDM2 mRNA in the cytoplasm, *k*_T_ is the translation rate for MDM2 mRNA, and *k*_i_ is the rate of nuclear localization for MDM2. MDM2 autoubiquitination happens at the rate *d*_mn_ and MDM2 binds to ARF at the rate *k*_3_. Lastly, ARF is produced at the rate *k*_a_ and degraded at the rate *d*_a_. The binding properties of p53 and the MDM2 promoter have been investigated experimentally by Weinberg et al. (2005), who showed that the appropriate Hill coefficient for the Hill function is 1.8.

A list of the values used for these parameters can be found in Table 2. The initial conditions were chosen by letting the system run until it settled into a stable limit cycle and then by using the values for the time when nuclear MDM2 levels were at a maximum.

**Table 2:** **Parameters used in the model**.

Parameter	Description	Value	Value (alternate expression)
*k*_p_	p53 Production	0.5 proteins/s	8.30 × 10^−3^/nM s
*k*_1_	MDM2 dependent p53 degradation	9.963 × 10^−6^/s	6 × 10^−4^/nM s
*d*_p_	p53 Decay	1.925 × 10^−5^/s	10 h half-life
*k*_m_	p53-Independent MDM2 production	1.5 × 10^−3^ RNA/s	1 RNA per 666 s
*k*_2_	p53-Dependent MDM2 production	1.5 × 10^−2^/s	Maximum of 1 RNA per 66 s
*K*_D_	Dissociation constant	740 proteins	12.3 nM
*k*_0_	RNA transport from nucleus to cytoplasm	8.0 × 10^−4^/s	14.4 min for half the proteins to move
*d*_rc_	MDM2 mRNA decay in cytoplasm	1.444 × 10^−4^/s	1 h 20 min half-life
*k*_T_	Transcription rate	1.66 × 10^−2^ proteins/s	One protein per RNA per min
*k*_i_	Protein transport from cytoplasm to nucleus	9.0 × 10^−4^/s	12.4 min for half the proteins to move
*d*_mn_	MDM2 autoubiquitination	1.66 × 10^−7^/s	2.76 × 10^−9^/nM s
*k*_a_	ARF production	0.5 proteins/s	8.30 × 10^−3^/nM s
*d*_a_	ARF decay	3.209 × 10^−5^/s	6 h half-life
*k*_3_	MDM2-ARF complex formation rate	9.963 × 10^−6^/s	6 × 10^−4^/nM s

**Figure 4:**
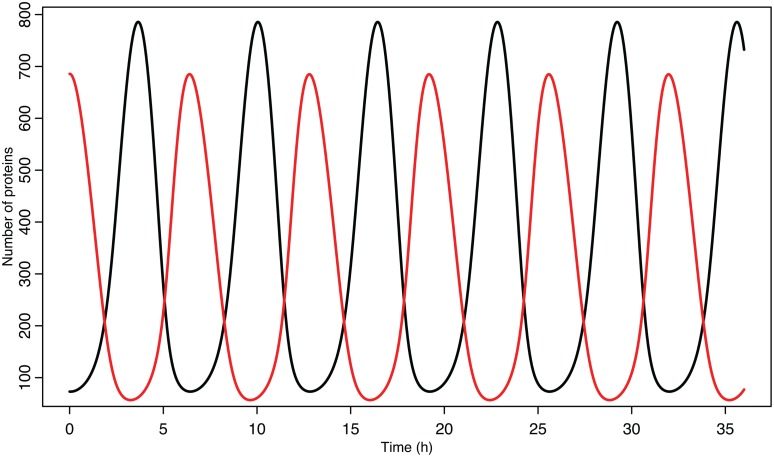
**p53 and MDM2 oscillating in the deterministic model**. p53 is in black, MDM2 is in red.

Experimental observations of the p53-MDM2 feedback loop have found periods of oscillations between 4 and 7 h (Geva-Zatorsky et al., 2006, 2010). Due to scarcity of experimentally verified data, most of parameters in the model were chosen by hand in order to produce oscillations with a similar period. Some of the parameters were constrained by experimental data. *K*_D_ was found to be 12.3 nM by Weinberg et al. (2005). Some experimental results suggested that the half-life for MDM2 mRNA should be in the range of 1–2 h (Hsing et al., 2000; Mendrysa et al., 2001), so this constrained our choice of the decay rate. The MDM2 translation rate, *k*_T_, was assumed to be one protein per mRNA molecule per minute, approximately the value estimated by Cai and Yuan (2009). The transport rate for MDM2 mRNA was constrained to be in the range of 5–40 min, based on Mor et al. (2010). The half-life of the ARF protein, *d*_a_, was chosen to be 6 h based on Kuo et al. (2004). Complex formation rates were assumed to be 6 × 10^−4^/nM s, a reasonable rate for protein–protein interactions (Northrup and Erickson, 1992). It was further assumed that the p53-MDM2 interaction would always result in p53 degradation. MDM2-independent p53 turnover was assumed to give a half-life of 10 h for the p53 protein; this is essentially negligible in this model, but this term was included in the model so that a bifurcation value could be calculated for it. Cytoplasmic volume was assumed to be 1000 μm^3^ with a nuclear volume of 100 μm^3^. The values for p53 production, ARF production, basal MDM2 mRNA production, p53 induced MDM2 mRNA production, MDM2 nuclear import, and MDM2 autoubiquitination were unknown. These unknown parameters were chosen manually in order to produce oscillations similar to the ones observed in experiments on single cells. Although only one set of parameters was produced for this model, the choice is certainly not unique given the somewhat loose selection criteria. The model produces oscillations with a period of 6.4 h as can be seen in Figure 4. Bifurcation points for the model are listed in Table 3. The bifurcation points were found numerically using Matlab (MathWorks, Inc.).

**Table 3:** **Bifurcation points in the deterministic model**.

Parameter	Bifurcation value	Oscillatory behavior
*k*_p_	0.215/s	Undamped: 0.215 ≤ *k*_p_ ≤ 1.462
*k*_p_	1.462/s	Damped: *k*_p_ ≤ 0.215 ∪ *k*_p_ ≥ 1.462
*k*_1_	2.903 × 10^−6^/s	Undamped: 2.903 × 10^−6^ ≤ *k*_1_ ≤ 1.834 × 10^−5^
*k*_1_	1.834 × 10^−5^/s	Damped: *k*_1_ ≤ 2.903 × 10^−6^ ∪ *k*_1_ ≥ 1.834 × 10^−5^
*d*_p_	4.237 × 10^−4^/s	Undamped: *d*_p_ ≤ 4.237 × 10^−4^
*k*_m_	2.788 × 10^−3^/s	Undamped: *k*_m_ ≤ 2.788 × 10^−3^
*k*_2_	7.501 × 10^−3^/s	Undamped: 7.501 × 10^−3^ ≤ *k*_2_ ≤ 0.118
*k*_2_	0.118/s	Damped: *k*_2_ ≤ 7.501 × 10^−3^ ∪ *k*_2_ ≥ 0.118
*K*_D_	253.083	Undamped: 253.083 ≤ *K*_D_ ≤ 1723.058
*K*_D_	1723.058	Damped: *K*_D_ ≤ 253.083 ∪ *K*_D_ ≥ 1723.058
*k*_0_	7.010 × 10^−6^/s	Undamped: 7.010 × 10^−6^ ≤ *k*_0_ ≤ 6.160 × 10^−3^
*k*_0_	6.160 × 10^−3^/s	Damped: *k*_0_ ≤ 7.010 × 10^−6^ ∪ *k*_0_ ≥ 6.160 × 10^−3^
*d*_rc_	8.714 × 10^−5^/s	Undamped: 8.714 × 10^−5^ ≤ *d*_rc_ ≤ 2.704 × 10^−4^
*d*_rc_	2.704 × 10^−4^/s	Damped: *d*_rc_ ≤ 8.714 × 10^−5^ ∪ *d*_rc_ ≥ 2.704 × 10^−4^
*k*_T_	8.760 × 10^−3^/s	Undamped: 8.760 × 10^−3^ ≤ *k*_T_ ≤ 2.936 × 10^−2^
*k*_T_	2.936 × 10^−2^/s	Damped: *k*_T_ ≤ 8.760 × 10^−3^ ∪ *k*_T_ ≥ 2.936 × 10^−2^
*k*_i_	6.845 × 10^−6^/s	Undamped: 6.845 × 10^−6^ ≤ *k*_i_ ≤ 1.559 × 10^−2^
*k*_i_	1.559 × 10^−2^/s	Damped: *k*_i_ ≤ 6.845 × 10^−6^ ∪ *k*_i_ ≥ 1.559 × 10^−2^
*d*_mn_	1.251 × 10^−6^/s	Undamped: *d*_mn_ ≤ 1.251 × 10^−6^
*k*_a_	0.324/s	Undamped: 0.324 ≤ *k*_a_ ≤ 0.963
*k*_a_	0.963/s	Damped: *k*_a_ ≤ 0.324 ∪ *k*_a_ ≥ 0.963
*d*_a_	2.088 × 10^−3^/s	Undamped: *d*_a_ ≤ 2.088 × 10^−3^
*k*_3_	5.866 × 10^−6^/s	Undamped: *k*_3_ ≥ 5.866 × 10^−6^

### Stochastic simulation algorithm

The Gillespie algorithm is one of the most commonly used methods of stochastic simulation (Gillespie, 1977). The Gillespie algorithm has the advantage of being exact, unfortunately, it is also computationally expensive. In order to conduct our investigation we chose to instead use an approximate simulation, because the Gillespie algorithm is too slow for the required complexity and number of simulation runs.

The algorithm we created was based on the concepts of a finite difference integrator. In a finite difference integrator a system of differential equations is evaluated by first calculating each of the derivatives at a point in time, then multiplying them by the time step size, and finally updating each of the variables by the corresponding amount. In our algorithm, rather than being evaluated as a single set of derivatives each chemical reaction is evaluated separately. When the simulation evaluates a chemical reaction, the first step is to use the law of mass-action and the average of the current chemical concentrations, and their concentrations after the last time the reaction was evaluated, to find an expectation value for the number of times the reaction will occur during this time step. Next, the expectation value for the number of times the reaction will occur is set as the expectation value for a Poisson random number generator and the result is the number of times the reaction will actually occur during that time step. This gives the algorithm a strong resemblance to the well known tau leap method (Gillespie, 2007), in which Poisson random numbers are used in combination with the Gillespie algorithm to improve efficacy. In order to improve efficiency while preserving accuracy in our algorithm, an adaptive time step is used. The program evaluates each reaction 0.5*^N^* times per simulated second, with *N* chosen such that the expectation value for a particular evaluation of a reaction is lower than a preset threshold multiplied by the quantity of the chemical molecules involved. In this way parts of the system that are changing rapidly are evaluated with a low enough time step to prevent numerical errors, without needing to waste additional computations on the slower reactions.

**Figure 5:**
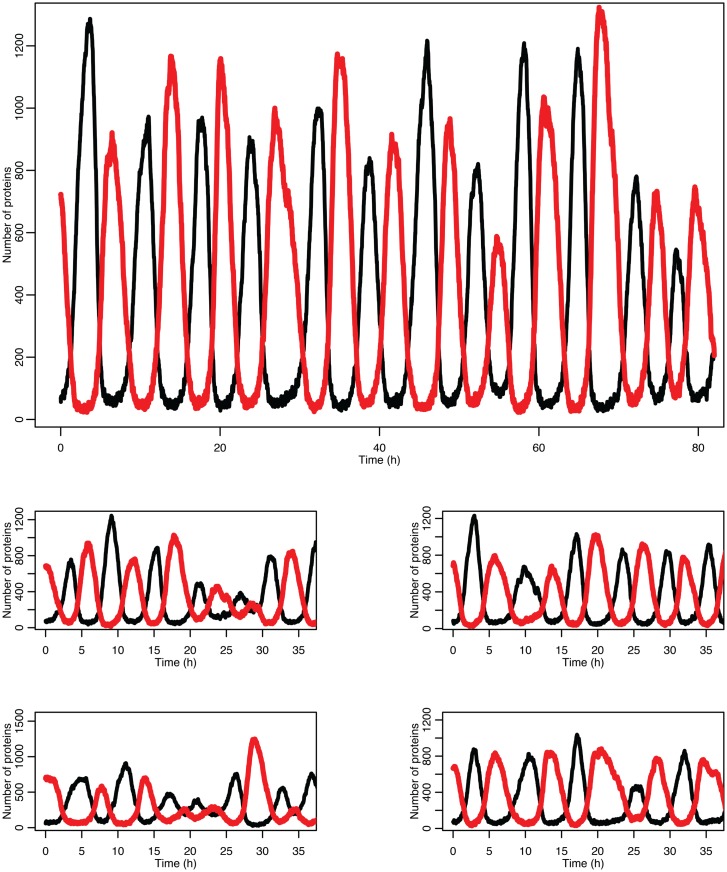
**Examples of time courses in the stochastic model**. p53 is in black and MDM2 is in red.

Figure 5 shows some examples of individual simulation runs for this model. The stochastic nature of the simulation leads to a number of interesting differences arising from the desynchronization of the individual model runs as well as from applying a distribution of p53 values into the non-linear function for MDM2 production.

## Results

### Desynchronization in general

In order to understand how the individual stochastic realizations of our model fall out of synch with each other let us first consider how stochastic systems may fall out of synchronization in general. An experiment averaging protein levels across many cells is analogous to looking at the average of many runs of a stochastic system. As such, it is interesting to consider how aggregate average behavior differs from the behavior of individual model runs. A given run of the stochastic model will not necessarily just be equal to the deterministic model plus noise. At any given step the stochastic model’s variables depend on the values of the variables at the previous time step. For a periodic model this will result not only in noise moving variables up and down but also in random stepping forwards and backwards of the model’s phase. Consequently, an ensemble of model runs will fall out of synchronization over time. Imagine for simplicity a stochastic model based on a deterministic model with a variable given by Asin(ωt+φ). In the stochastic model random chance continuously moves each run in the ensemble toward or away from the next peak. Considering the central limit theorem applied over a large number of runs, one would then expect the distribution of timing of the peak in individual runs to approach a normal distribution. If all the runs are initialized from the same starting point, then the amplitude of the mean will not be Asin(ωt+φ) but rather it will be

$$A\int_{-\infty }^{\infty }\frac{1}{\sigma \sqrt{2\pi }}e^{-\frac{1t^{\prime 2}}{2\sigma^{2}}}\sin\left(\omega t+\varphi +\omega t^{\prime }\right)dt^{\prime }$$

because the timing of each run will be shifted with a Gaussian weighting given to the shift. Since the width of the distribution will increase proportionally to the square root of time, the standard deviation σ can be expanded as $\alpha \sqrt{t}$, where α is a parameter related to the rate of desynchronization. This integral then works out to be

$$Ae^{-\frac{\omega^{2}\alpha^{2}t}{2}}\sin\left(\omega t+\varphi \right)$$

Consider a 2π periodic function that is integrable on the interval from −π to π. This function could be expressed as a Fourier series such that

$$f\left(t\right)=\frac{a_{0}}{2}+\overset{\infty }{\underset{n=1}{\sum }}\left[a_{n}\cos\left(nt\right)+b_{n}\sin\left(nt\right)\right]$$

or equivalently

$$f\left(t\right)=\frac{a_{0}}{2}+\overset{\infty }{\underset{n=1}{\sum }}\left[a_{n}\sin\left(nt+\frac{\pi }{2}\right)+b_{n}\sin\left(nt\right)\right]$$

Applying the result above we find that the function will be changed by desynchronization to become

$$f^{\prime }\left(t\right)=\frac{a_{0}}{2}+\overset{\infty }{\underset{n=1}{\sum }}\left[a_{n}\sin\left(nt+\frac{\pi }{2}\right)+b_{n}\sin\left(nt\right)\right]e^{-\frac{n^{2}\alpha^{2}t}{2}}$$

Since the decay is proportional to the square of the frequency, any function will rapidly take on the appearance of a single decaying sine-function curve as time progresses.

### Desynchronization in the stochastic model

**Figure 6:**
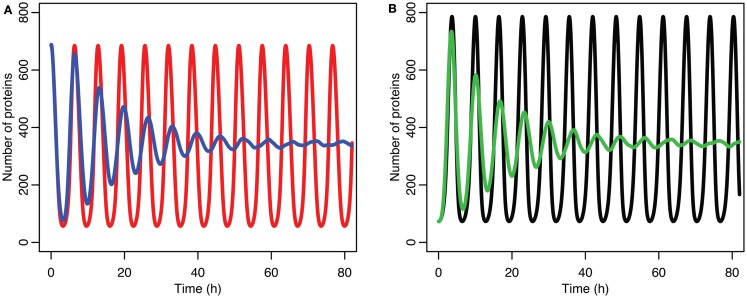
**Comparison of stochastic and deterministic models**. **(A)** Shows the comparison for MDM2 with MDM2 from the deterministic model in red and from the mean of 5,000 runs of the stochastic model in blue. **(B)** Shows the comparison for p53 with the deterministic model in black and from the mean of 5,000 runs of the stochastic model in green.

**Figure 7:**
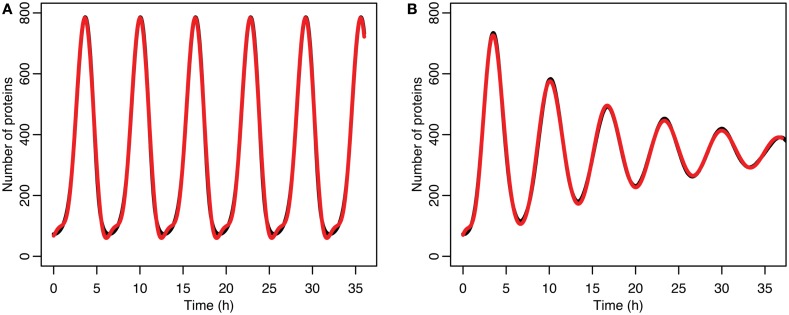
**(A)** Comparison of p53 levels in the deterministic model in black to a curve fitted to it from the function $f\left(t\right)=a_{0}+a_{1}\sin\left(\omega t+\varphi_{1}\right)+a_{2}\sin\left(\text{2}\omega t+\varphi_{2}\right)$ in red. **(B)** Comparison of p53 levels in the stochastic model in black to a curve fitted to it from the function $f\left(t\right)=a_{0}+e^{-\frac{\alpha^{2}t}{2}}a_{1}\sin\left(\omega t+\varphi_{1}\right)+e^{-\frac{\text{4}\alpha^{2}t}{2}}a_{2}\sin\left(\text{2}\omega t+\varphi_{2}\right)$ in red.

The damping caused by desynchronization in the stochastic model can be seen in Figure 6. The deterministic and stochastic systems can be compared by fitting a curve to the time series for p53. Specifically:

$$f\left(t\right)=a_{0}+a_{1}\sin\left(\omega t+\varphi_{1}\right)+a_{2}\sin\left(\text{2}\omega t+\varphi_{2}\right)$$

for the deterministic model, and

$$f\left(t\right)=a_{0}+e^{-\frac{\alpha^{2}t}{2}}a_{1}\sin\left(\omega t+\varphi_{1}\right)+e^{-\frac{\text{4}\alpha^{2}t}{2}}a_{2}\sin\left(\text{2}\omega t+\varphi_{2}\right)$$

for the stochastic model. Table 4 lists the parameter estimates for the deterministic model as well as 95% confidence intervals for the stochastic model. Figure 7 shows graphs of the functions and their best fits. The best fit was determined by using least squares regression on the mean p53 values from 5,000 instances of the stochastic model. The upper and lower bounds were found by using bootstrapping on the 5,000 instances that were used to compute the best fit. The 95% confidence intervals for the amplitude and phase of the second sine curve ended up being very large due to the curve fitting function jumping between local minima. To ensure that the algorithm was being run at a high enough numerical precision, an additional 5,000 instances were generated with the acceptable error parameter in the code selected to equal 10 times the value used in this analysis. The resulting new confidence intervals were compared to the ones from the higher accuracy runs. In all cases significant overlap of the intervals was found, suggesting that the acceptable error was set low enough in the high accuracy runs to result in only negligible deviations from an exact solution.

**Table 4:** **Comparisons of the parameters found when fitting the deterministic model’s p53 levels to the function $f\left(t\right)=a_{0}+a_{1}\sin\left(\omega t+\varphi_{1}\right)+a_{2}\sin\left(\text{2}\omega t+\varphi_{2}\right)$ and the stochastic model’s p53 levels to the function $f\left(t\right)=a_{0}+e^{-\frac{\alpha^{2}t}{2}}a_{1}\sin\left(\omega t+\varphi_{1}\right)+e^{-\frac{\text{4}\alpha^{2}t}{2}}a_{2}\sin\left(\text{2}\omega t+\varphi_{2}\right)$**.

	Parameters fitted to deterministic model	Parameters fitted to stochastic model	Lower bound for stochastic parameters	Upper bound for stochastic parameters
α	N/A	21.8/s^1/2^	21.2/s^1/2^	22.5/s^1/2^
ω	2.73 × 10^−4^/s	2.63 × 10^−4^/s	2.62 × 10^−4^/s	2.64 × 10^−4^/s
*A*_0_	332	346	345	347
*A*_1_	−348	−396	−406	−388
*f*_1_	1.21	1.40	1.38	1.43
*A*_2_	105	136	−136	144
*f*_2_	0.633	−1.16	−36.6	13.6

**Figure 8:**
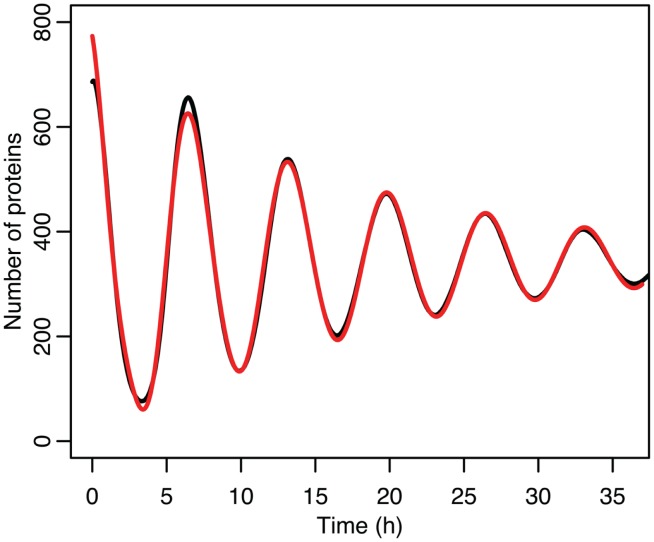
**Comparison of nuclear MDM2 levels in the stochastic model in black to a curve fitted to it from the function $f\left(t\right)=a_{0}+e^{-\frac{\alpha^{2}t}{2}}a_{1}\sin\left(\omega t+\varphi_{1}\right)+e^{-\frac{4\alpha^{2}t}{2}}a_{2}\sin\left(2\omega t+\varphi_{2}\right)$ in red**.

The differences between the stochastic model’s behavior and the deterministic model’s behavior are statistically significant. Most striking is that the frequency of the oscillations was changed by stochastic effects. The same analysis has been done on nuclear MDM2 levels, which can be seen in Figure 8 and Table 5. The discrepancy between the fitted curve for MDM2 levels and the levels from the simulation hints at another difference between stochastic and deterministic systems, which will be discussed below. It is also worth noting that this stochastic model only considers the differences between cells due to noise in a few chemical reactions. In a real cell there would be many more factors contributing to desynchronization. Even simply adding mRNA for the p53 and ARF included in this model raises the desynchronization parameter a from 21.8 to 23.5 s^−1/2^ (a mean of 30 mRNA molecules was used for this simulation). Additionally, differences in cell volume would increase desynchronization by altering protein concentrations between cells.

**Table 5:** **Comparisons of the parameters found when fitting the deterministic model’s nuclear MDM2 levels to the function $f\left(t\right)=a_{0}+a_{1}\sin\left(\omega t+\varphi_{1}\right)+a_{2}\sin\left(\text{2}\omega t+\varphi_{2}\right)$ and the stochastic model’s nuclear MDM2 levels to the function $f\left(t\right)=a_{0}+e^{-\frac{\alpha^{2}t}{2}}a_{1}\sin\left(\omega t+\varphi_{1}\right)+e^{-\frac{\text{4}\alpha^{2}t}{2}}a_{2}\sin\left(\text{2}\omega t+\varphi_{2}\right)$**.

	Parameters fitted to deterministic model	Parameters fitted to stochastic model	Lower bound for stochastic parameters	Upper bound for stochastic parameters
α	N/A	20.7/s^1/2^	20.2/s^1/2^	21.3/s^1/2^
ω	2.73 × 10^−4^/s	2.63 × 10^−4^/s	2.62 × 10^−4^/s	2.63 × 10^−4^/s
*A*_0_	302	345	343	346
*A*_1_	−314	−372	−379	−365
*f*_1_	−1.72	−1.49	−1.51	−1.47
*A*_2_	−71	−78.5	−82.8	−74.6
*f*_2_	−1.73	−0.80	−0.87	−0.73

### Changes due to non-linear effects

**Figure 9:**
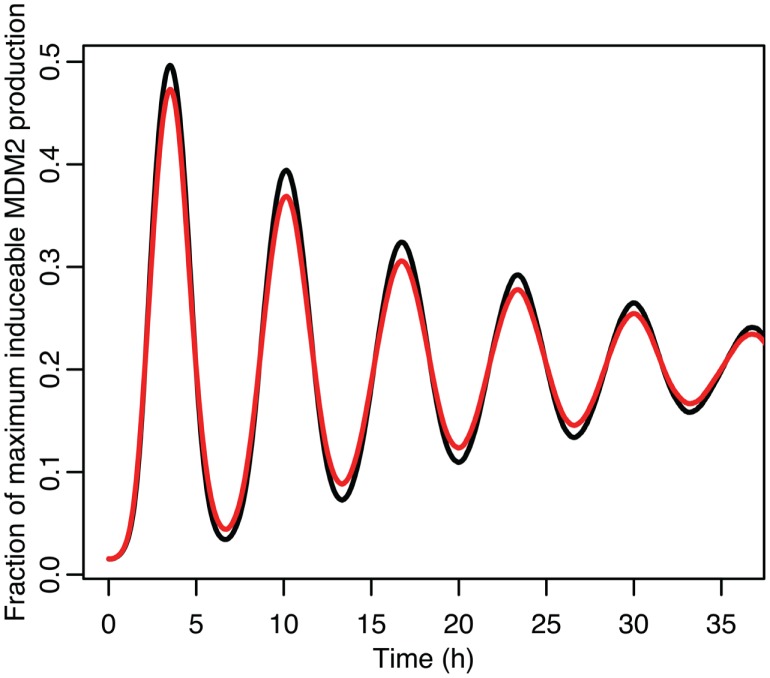
**A comparison of the function $\frac{{\left[p53\right]}^{1.8}}{k_{\text{d}}^{1.8}+{\left[p53\right]}^{1.8}}$ between the function applied to mean p53 values in black and the mean of the function when applied to the distribution of p53 values in red**.

The mean of a stochastic ensemble for the stochastic model deviates from the deterministic model not just from desynchronization but also due to non-linear effects. For a non-linear function applied to a distribution of inputs, the mean of the function will not necessarily be equal to the function of the mean. In other words, as is well known in statistics, the following is usually true: <*f*(*x*)> ≠ *f*(<*x*>), unless *f* is a linear function of *x*. Production of MDM2 mRNA in this model is clearly non-linear because it is proportional to $f(\text{p}53)=\frac{{\left[\text{p}53\right]}^{1.8}}{k_{\text{d}}^{1.8}+{\left[\text{p}53\right]}^{1.8}}.$ Figure 9 compares the function of the mean to the mean of the function for this case. Mean MDM2 values in the stochastic model are determined by <*f*(p53)> (the red curve in Figure 9) which has a different amplitude then *f*(<p53>) (the black curve in Figure 9). This discrepancy causes the behavior of the system to change relative to the deterministic case, which only has mean p53 values. This is also the most likely source of the discrepancy between the fitted curve in Figure 8 and the actual levels of MDM2. With production that behaves differently, the initial conditions in the simulation would not have represented a point on the limit cycle for MDM2 levels. As a consequence, the system would have been moving toward the limit cycle at the same time as it was desynchronizing. The simple fitted curve cannot possibly account for this, which is why it did not fit well. p53 levels would also have been affected by this but this does not seem to have been a large enough effect to be readily noticeable on the graph.

**Figure 10:**
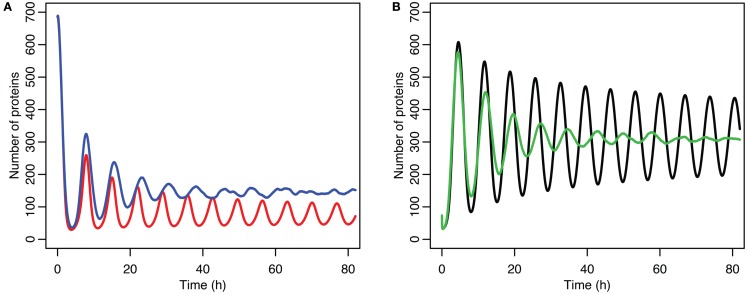
**Comparison of stochastic and deterministic models when p53 production is near the lower bifurcation point**. **(A)** Shows the comparison for MDM2 with MDM2 from the deterministic model in red and from the mean of 5,000 runs of the stochastic model in blue. **(B)** Shows the comparison for p53 with the deterministic model in black and from the mean of 5,000 runs of the stochastic model in green.

**Figure 11:**
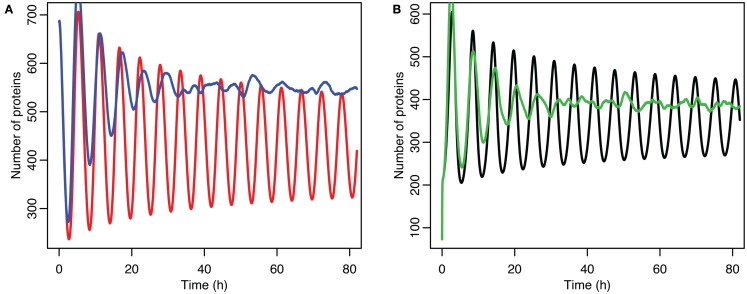
**Comparison of stochastic and deterministic models when p53 production is near the upper bifurcation point**. (**A**) Shows the comparison for MDM2 with MDM2 from the deterministic model in red and from the mean of 5,000 runs of the stochastic model in blue. (**B**) Shows the comparison for p53 with the deterministic model in black and from the mean of 5,000 runs of the stochastic model in green.

Although the effect on the amplitude of the oscillations with the original parameters was relatively small, amounting to approximately 5%, the non-linear effects can be larger in other situations. Consider the case when the p53 production rate is set near to the lower bifurcation point, as shown in Figure 10. In this case the mean level of MDM2 from the stochastic model ends up being higher than the maximum amplitude of the oscillations in the deterministic model. A similar phenomenon occurs when p53 production is near the upper bifurcation point as is shown in Figure 11.

### Excursions from the mean

**Figure 12:**
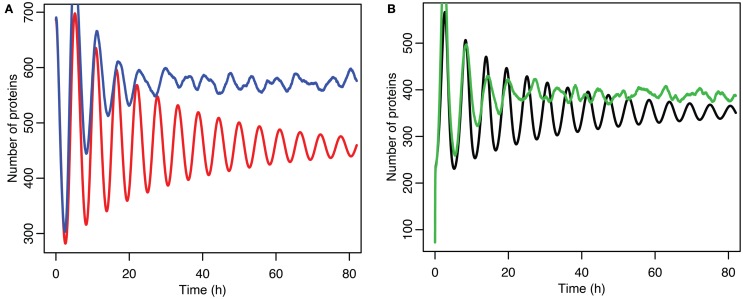
**Comparison of stochastic and deterministic models when the p53 production value is past the upper bifurcation point**. **(A)** Shows the comparison for MDM2 with MDM2 from the deterministic model in red and from the mean of 5,000 runs of the stochastic model in blue. **(B)** Shows the comparison for p53 with the deterministic model in black and from the mean of 5,000 runs of the stochastic model in green.

**Figure 13:**
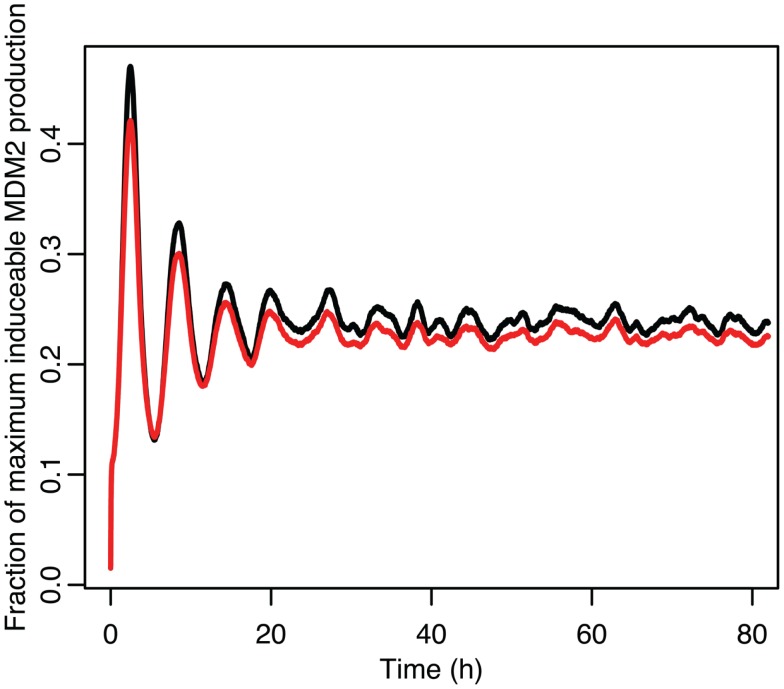
**Comparison of the function $\frac{{\left[p53\right]}^{1.8}}{k_{\text{d}}^{1.8}+{\left[p53\right]}^{1.8}}$ between the function applied to mean p53 values in black and the mean of the function when applied to the distribution of p53 values in red**.

**Figure 14:**
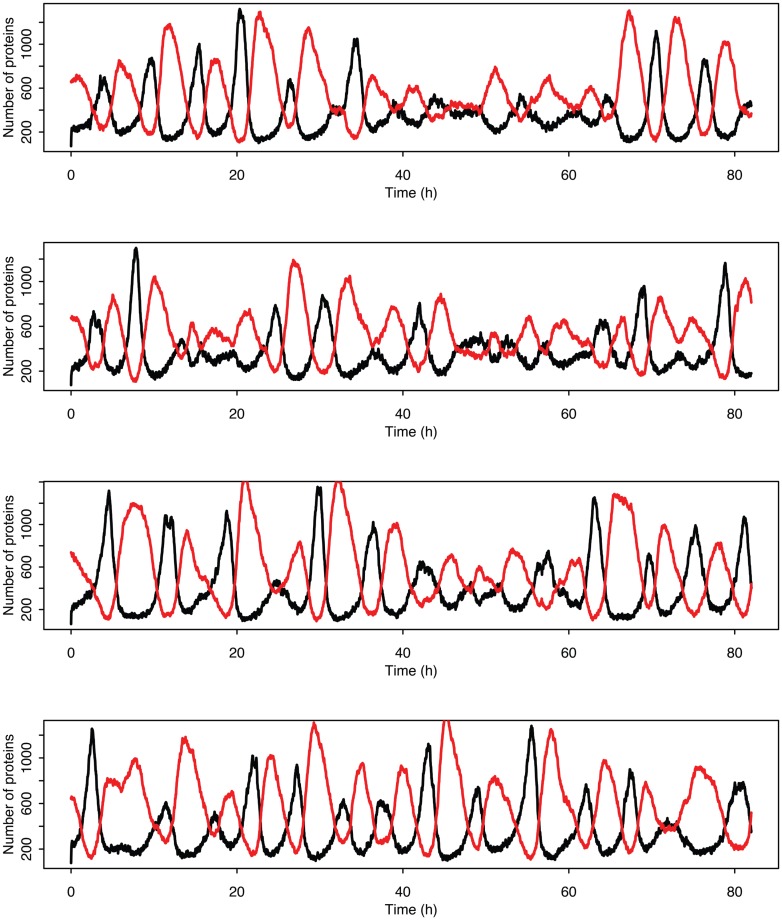
**Examples of individual stochastic realizations when p53 production is past the upper bifurcation point**. p53 is in black MDM2 is in red.

Stochastic effects continue to play an interesting role in the system’s behavior even as we move past the upper bifurcation point, so that the deterministic model exhibits damped oscillations. For Figures 12–14, p53 production was set to 1.6, putting the system into the realm of damped oscillations. In Figure 12 we can see that as the oscillations decay, the MDM2 levels settle in at a value significantly higher in the stochastic model than the deterministic one. From Figure 13 we can see that the non-linear effects of variable p53 levels are still altering behavior, but something more is occurring this time. In Figure 12B we see that mean p53 levels are settling in at a level higher in the stochastic model than in the deterministic one. This seems strange in light of the higher MDM2 levels but Figure 14 shows the reason. The stochastic nature of the system is sufficient to cause significant excursions from the mean even though the oscillations should be decaying. Some of the oscillations that occur later on are even larger than the initial pulse. Similar behavior has been observed in other stochastic models such as the one presented in McKane and Newman (2005), but has not been previously observed in a model of the p53 system.

## Discussion

The stochastic work we present in this paper differs from previous modeling efforts in that its goal is primarily to compare the behavior of stochastic and deterministic realizations of the same model. This requires only a simple model; therefore much of the complexity of the p53 system can be ignored. Since the model presented in this work is not aimed at addressing DNA repair, or dealing with the problem of variable damage being done, it does not include such systems. The model presented here also differs from the previous models in a few other ways. Unlike in other models, MDM2 autoubiquitination was assumed to happen at a rate proportional to the square of MDM2 concentration. Given that MDM2 forms heterodimers with MDMX (Sharp et al., 1999), that MDMX inhibits MDM2 autoubiquitination (Okamoto et al., 2009), and that MDM2 ubiquitinates MDMX (De Graaf et al., 2003), it seems likely that one MDM2 molecule is ubiquitinating a second MDM2 molecule.

The work on the deterministic and stochastic models presented here demonstrates that the effects of stochasticity on the behavior of genetic regulatory networks cannot be dismissed without careful consideration. In our system stochastic effects altered every aspect of system behavior. In addition to desynchronization leading to the appearance of decaying oscillations, the amount of MDM2 in the system increased and the period of the oscillations changed. The changes in MDM2 levels became more obvious when p53 production was near bifurcation points. When the system was put into a state with decaying oscillations, the quantity of MDM2 still remained above that in the deterministic model, showing that stochasticity still alters behavior as the system is near a steady state. Furthermore, stochastic systems will not necessarily undergo damped oscillations even when assigned parameters that would cause damped oscillations in a deterministic system. Instead, they may show sporadic oscillation-like excursions from the mean behavior. It would seem then that even for cells in a steady state, the distribution of protein levels across a population and over time could wreak havoc with attempts to model cell behavior. This has implications for researchers wishing to model cell-level processes, as systematic errors could occur in deterministic models with no obvious way to compensate for them. As computers and algorithms improve, it may be the case that simply moving to stochastic modeling of cell populations will become the most practical solution.

The demonstration that stochasticity can be relevant is very general, but it was also shown that the magnitude of the effects could vary significantly between systems. The effect on mean protein levels could be around 5%, as in the original parameter set, or around 50%, as in some of the parameter sets with differing p53 levels. The obvious way to experimentally test the relevance of stochasticity on any given system is by comparing data from cell populations to data from individual cells. Such experimental comparisons were, after all, the inspiration for investigating stochasticity in this system in the first place. The difference between a stochastic model and a deterministic one with different parameters are not likely to be obvious from population data, even if the effects of stochasticity are expected to be large. Testing the details of stochastic models will require investigating the behavior of individual cells. Of course, stochasticity is not the only factor that could drive individual cells to different behaviors. Factors such as differences in cell size, different cell cycle stages, and non-uniform distributions of components in cell culture medium could all alter behavior on the scale of single cells. Untangling these effects is potentially a fruitful area for future research.

A valuable way of expanding the utility of our model would be to link it to other models of related processes. The DNA repair and damage detection modules in Ma et al. (2005) would be a good example of this. Once one system is sufficiently well understood, it would be possible to begin analyzing how altering it changes connected systems, or conversely, how changing connected systems alters it. This could allow one to study downstream drug effects. For that kind of work it would likely be best to start as far upstream as possible, in order to facilitate the experimental control of inputs. For example, for the p53 system it would make sense to start with a model that quantifies the damage ionizing radiation causes to DNA and other cellular systems, because the level of radiation a cell is exposed to can be controlled in the lab. Then, once that is modeled accurately, one could study the DNA damage detection systems, and finally the p53 response. Repeating this process for other forms of damage, like for example ultraviolet light, could bring insight into the system’s behavior.

## Conflict of Interest Statement

The authors declare that the research was conducted in the absence of any commercial or financial relationships that could be construed as a potential conflict of interest.
